# The Relationship Between Images Posted by New Mothers on WeChat Moments and Postpartum Depression: Cohort Study

**DOI:** 10.2196/23575

**Published:** 2020-11-30

**Authors:** Weina Zhang, Lu Liu, Qijin Cheng, Yan Chen, Dong Xu, Wenjie Gong

**Affiliations:** 1 XiangYa School of Public Health Central South University Changsha China; 2 Changsha Hospital for Maternal and Child Health Care Changsha China; 3 Department of Social Work The Chinese University of Hong Kong Hong Kong SAR Hong Kong China; 4 Global Health and Health Systems School of Health Management Southern Medical University Guangzhou China; 5 Institute of Applied Health Research University of Birmingham Birmingham United Kingdom; 6 Department of Psychiatry University of Rochester Medical Center Rochester, NY United States

**Keywords:** social media, WeChat, WeChat Moments, postpartum depression

## Abstract

**Background:**

As social media posts reflect users’ emotions, WeChat Moments, the most popular social media platform in China, may offer a glimpse into postpartum depression in the population.

**Objective:**

This study aimed to investigate the features of the images that mothers posted on WeChat Moments after childbirth and to explore the correlation between these features and the mothers' risk of postpartum depression.

**Methods:**

We collected the data of 419 mothers after delivery, including their demographics, factors associated with postpartum depression, and images posted on WeChat Moments. Postpartum depression was measured using the Edinburgh Postnatal Depression Scale. Descriptive analyses were performed to assess the following: content of the images, presence of people, the people’s facial expressions, and whether or not memes were posted on WeChat Moments. Logistic regression analyses were used to identify the image features associated with postpartum depression.

**Results:**

Compared with pictures of other people, we found that pictures of their children comprised the majority (3909/6887, 56.8%) of the pictures posted by the mothers on WeChat Moments. Among the posts showing facial expressions or memes, more positive than negative emotions were expressed. Women who posted selfies during the postpartum period were more likely to have postpartum depression (*P*=.003; odds ratio 2.27, 95% CI 1.33-3.87).

**Conclusions:**

The vast majority of mothers posted images conveying positive emotions during the postpartum period, but these images may have masked their depression. New mothers who have posted selfies may be at a higher risk of postpartum depression.

**Trial Registration:**

International Clinical Trials Registry Platform ChiCTR-ROC-16009255; http://www.chictr.org.cn/showproj.aspx?proj=15699

## Introduction

In recent years, social media has become increasingly popular as a means for users to express their feelings and thoughts. Posts on social media platforms such as Facebook and Twitter can therefore serve as indicators or records of life events [1]. Worldwide, the number of monthly active users on Facebook and Twitter exceeds 2.6 billion and 330 million, respectively [2,3]. In China, WeChat is the most popular social media platform, with more than 1.15 billion active monthly users [4]. Based on data from both the National Bureau of Statistics of China and Tencent’s 2016 report [5,6], it is estimated that 72% of Chinese women (ages 15-80 years) use WeChat. Indeed, 60% of active users log into their WeChat accounts and post status updates with friends on WeChat Moments regularly [7]. Content posted on WeChat Moments is only visible to friends who follow one another. This social media platform is more private than Facebook and Twitter, for example [8]. Users are therefore more inclined to express their emotions authentically [8].

Some studies have used data collected from social media for sentiment analysis and have indicated that sentiment analysis has practical applications in many fields (eg, health care, finance, media, consumer markets, and government) and can facilitate the delivery of targeted information to people in said fields [9,10]. A review including 48 studies relevant to mental health issues used datasets from social media networks to research depression [11]. De Choudhury’s [12,13] studies predicted the onset of postpartum depression and significant postpartum changes in mothers by analyzing shared Facebook and Twitter data. However, the review indicated that most related studies have focused on Western countries and have relied on text-based analyses [11]. The authors therefore suggested that future research should include more technical analyses, such as image-based analyses. Images posted on social media can be important information carriers as they contain large volumes of data. For example, photos posted on Instagram by depressed individuals are more likely to be bluer, grayer, and darker [14]. Furthermore, postpartum depression and maternal sensitivity have been associated with a lower proportion of photos of mothers’ smiling babies [15], whereas selfies have been associated with a higher incidence of depression [16]. Certain facial expressions have been linked to depression and anxiety in social media users [17]. Theoretically, images posted on social media could be used to identify meaningful patterns in users’ mental health. The initial basic function of WeChat Moments, the social media platform with the most traffic in China, is to upload images. However, due to the privacy settings of WeChat Moments, image data are difficult to obtain. To our knowledge, apart from our previous work in which we used WeChat Moments text emojis as features that were then modeled for sentiment analysis of perinatal depression [18], no psychiatric study has used WeChat Moments to date.

In China, new mothers are expected to abide by a custom referred to as “doing the month,” during which they stay home for about 45 days after childbirth and refrain from contacting people frequently [19]. This makes screening for postpartum depression particularly challenging. However, WeChat Moments posts offer a novel method to study the emotions of new mothers in China. Although postpartum depression screening currently relies heavily on self-reported questionnaires, some studies have found that people with depression are inclined to hide their symptoms of depression [20]. Moreover, self-reported screening methods are susceptible to social desirability response bias [21], which may cause false negative results during postpartum depression screening. Given the challenges inherent to the self-reported questionnaire approach, WeChat Moments posts could provide a new avenue for postpartum depression screening. Our cohort study was designed to analyze the features of the images that new mothers posted on WeChat Moments and to explore the correlation between these features and the mothers' relative risk of postpartum depression.

## Methods

### Participant Recruitment

Participants were recruited from two maternity and childcare centers in the cities of Changsha and Yiyang in Hunan province, China. We recruited women in the obstetrics clinics of these two centers from September 2016 to February 2017. The following inclusion criteria were applied: women who were pregnant, aged ≥18 years, and had a gestation period of ≤13 weeks (pregnancy weeks were estimated based on the first day of the last menstrual period).

**Figure 1:**
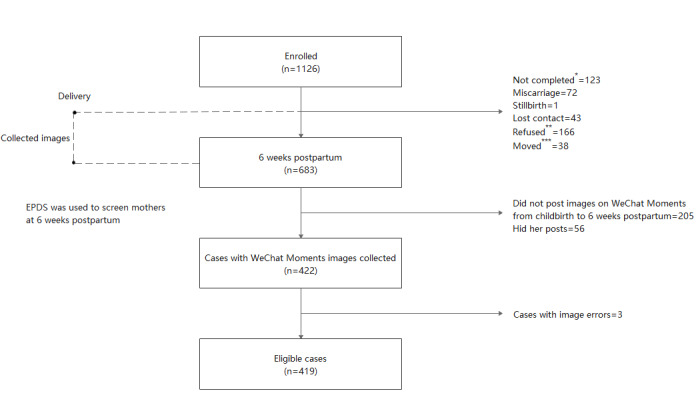
Participant recruitment and data collection process flow diagram. EPDS: Edinburgh Postnatal Depression Scale. *Mothers did not complete the EPDS at 6 weeks postpartum, **Mothers refused to participate in the investigation, ***Mothers moved to another city for childbirth and were unable to continue participating in the study.

The study was approved by the institutional review board of the Institute of Clinical Pharmacology of Central South University (ChiCTR-ROC-16009255). A total of 1126 women were recruited. In total, 15,647 WeChat Moments images and 6609 posts from 419 mothers, 123 of whom had postpartum depression, were used in this study. The participant recruitment and data collection process are depicted in Figure 1. The use of all the data from WeChat Moments in this study was authorized by the users.

### Measures

A number of different tools were used to collect data. We used questionnaires to measure the participants’ perinatal depression levels and other psychological factors from the first trimester to 6weeks postpartum and collected the mothers’ WeChat Moments data from childbirth to 6 weeks postpartum. The questionnaire was designed using simple items to investigate the participants’ general demographic characteristics including age, first pregnancy, monthly income, education, and history of depression. A token gift of 10 RMB (US $1.5) was given to each participant who completed a questionnaire.

We used the Pittsburgh Sleep Quality Index [22] to measure the sleep quality of the participants during the postpartum period. In this index, sleep quality is divided into different grades based on the total score, where 0-5 indicates very good sleep quality, 6-10 indicates good sleep quality, 11-15 indicates average sleep quality, and 16-21 indicates poor sleep quality.

We used the 7-item Generalized Anxiety Disorder Scale [23] to measure the anxiety of the participants during the postpartum period. A total score of 10 or higher on this scale is indicative of anxiety.

We collected the images that mothers had posted on WeChat Moments from childbirth to 6 weeks postpartum using the WebCrawler document assembler. The privacy settings allowing image visibility to online friends in WeChat Moments include “all,” “last six months,” “month,” and “three days” (friends will not be able to see the WeChat Moments earlier than the selected timeframe), so images were collected every 2 days.

The Edinburgh Postnatal Depression Scale (EPDS) [24,25] was used to screen for postpartum depression. The EPDS is a 10-item self-rated questionnaire. Each item is scored from 0 to 3, with a total score ranging from 0 to 30. The Chinese language EPDS used in this study was translated by Wang Yuqiong [26]. The critical value was 9.5.

### Image Preprocessing

For image analysis, the following 3 errors were preprocessed and removed: (1) images that returned link error messages or manifested incompletely, (2) images with a screen resolution less than 800x600, and (3) black images. The remaining eligible images were converted to readable formats such as JPG and PNG.

### Feature Extraction

Several different types of information were automatically extracted from the collected WeChat Moments data. Information categories included the following: post coloring, post volume and frequency, and the time posts were made.

#### Coloring

Colors can be expressed in various color spaces. In this study, we used the hue, saturation, and value color model. These 3 color properties are commonly used during image analysis [14,17]. Hue describes an image’s coloring on the light spectrum, with the color type falling between 0° and 360°. Lower hue values indicate redder colors, and higher hue values indicate bluer colors (eg, 0 is red, 60 is yellow). Saturation refers to the vividness of an image and ranges from 0 to 1. Low saturation makes an image appear gray and faded (eg, 0 represents no color and is a shade of gray). Value refers to the brightness of an image, which ranges from 0 to 1. Lower brightness scores indicate a darker image (eg, 0 represents black). The aforementioned colors of all the images were calculated in individual units. The mean number of pixels in each image was computed to determine the hue, saturation, and value. The colors of the images were extracted using MATLAB (version 9.7; MathWorks) and converted with OpenCV (version 4.0.0; Intel).

#### Volume and Frequency

The posting volume (each post contained 1-9 images) and the frequency of the WeChat Moments posts were calculated for each individual. Referencing Hicks’ and Brown’s [27] classification of the frequency of Facebook use, we defined the frequency of WeChat Moments posts as “high” (≥30 posts a month), “medium” (4-30 posts a month), and “low” (<4 posts a month).

#### Timing

Late-night posts were defined as posts made between 10 PM and 6 AM. Depending on whether the mother posted late at night or not, we defined this as “Yes” or “No,” respectively.

### Image Annotation

#### Assessing Agreement Among Researchers

To ensure the quality of our study, 11 independent researchers were recruited and uniformly trained to manually identify the content and emotions of the collected images. In line with Reece and Danforth’s study [14], each photo was categorized by 3 different researchers. The researchers were not given any information about the mothers who provided the images. When the researchers had divergent opinions about the images, the following 3 rules were applied: (1) if 2 of the 3 researchers were in agreement, we would use the majority opinion as the correct result; (2) if all the researchers disagreed on a particular image, the principal investigators would discuss and reevaluate the image; (3) if the researchers had objections regarding the aforementioned reevaluation result, the image was classified as “other.” In total, 4.7% (742/15,647) of the collected images were classified as “other.”

#### Labelling Image Content

Images posted on WeChat Moments can contain diverse content. Using the criteria of previous studies [14,17], we labeled all images using a system with 11 types of tags: (1) people, (2) objects (eg, bed, clothes), (3) animals (eg, dog, cat), (4) landscapes (eg, lakeside, prairie), (5) vehicles (eg, automobile, bus), (6) plants (eg, flower, tree), (7) food (eg, cake, noodles), (8) buildings (eg, apartment, office building), (9) memes, (10) WeChat Moments advertisements, and (11) others. Each image could only be labeled with 1 tag. When a picture had multiple components (eg, a person hugging a pet, a landscape with some people), the following 2 rules were applied: (1) if an image contained people and their facial expressions were clear, we used “people” as the tag to define the content of the image; (2) If the image did not contain a person, we would consider what the mother who posted the image was trying to express and then decide on the tag accordingly. We also calculated the total number of tags and the percentage of each tag type.

#### Characterization of Pictures of People

We used 2 measures to characterize the pictures of people. The first measure was the number and proportion of selfies (ie, close-up facial images or images of the same person appearing multiple times) and pictures of the mothers’ children. Studies have shown that posting selfies on social media can have adverse psychological effects on women [16,28]. Furthermore, a study by Schoppe-Sullivan et al [29] suggested that mothers with a high need for identity recognition were more likely to post pictures of their children on Facebook. Therefore, if mothers loaded selfies or pictures of their children, we placed them into a “yes” category. If they did not, we placed them into a “no” category. If mothers posted both selfies and pictures of their children, researchers assessed and categorized the images according to the subject or focus of the pictures. If researchers were in disagreement, images were processed according to the agreement assessment procedure we have described. The second measure was the number of positive (eg, joy and surprise) and negative (eg, anger, disgust, fear, and sadness) facial expressions of the persons in any given collected picture. Referencing the study by Dáu et al [15], which found that women who posted fewer images of smiling babies were more likely to exhibit maternal depressive symptoms, we categorized the facial expressions presented in each picture as positive or negative and then counted the proportion of each type of expression for each mother. Based on the proportion of positive facial expressions, we classified mothers as “No (0%);” “Yes, with a low proportion of positive facial expressions (0%-50%);” and “Yes, with a high proportion of positive facial expressions (>50%).” The first measure demonstrated the content of the pictures, while the second denoted the emotional expressions of the people in the pictures.

#### Assessment of Memes Posted to WeChat Moments

Through the expressions and words in the collected memes, we asked the researchers to categorize the mood of the memes as either positive or negative. We then calculated the number and percentage of positive and negative moods in the collected memes. However, we used only “Yes” and “No” classification criterion in the logistic regression model. Depending on whether or not mothers had posted memes, they were categorized as either a “Yes” or a “No.” If memes were posted, they were assigned a “Yes,” and if memes were not posted, they were assigned a “No.”

### Statistical Analysis

Different features of mothers with or without postpartum depression were analyzed using the chi-square test and *t* test. While controlling for the factors associated with postpartum depression, a binary logistic regression was conducted to explore the association between the image features and postpartum depression using the image features as the independent variables and with or without postpartum depression as the dependent variables. The statistical analyses were performed using SPSS (version 25; IBM). The classification of images in this study is based on previous studies [14-16,27-29].

## Results

### Demographic Characteristics of the Participants

**Figure 2:**
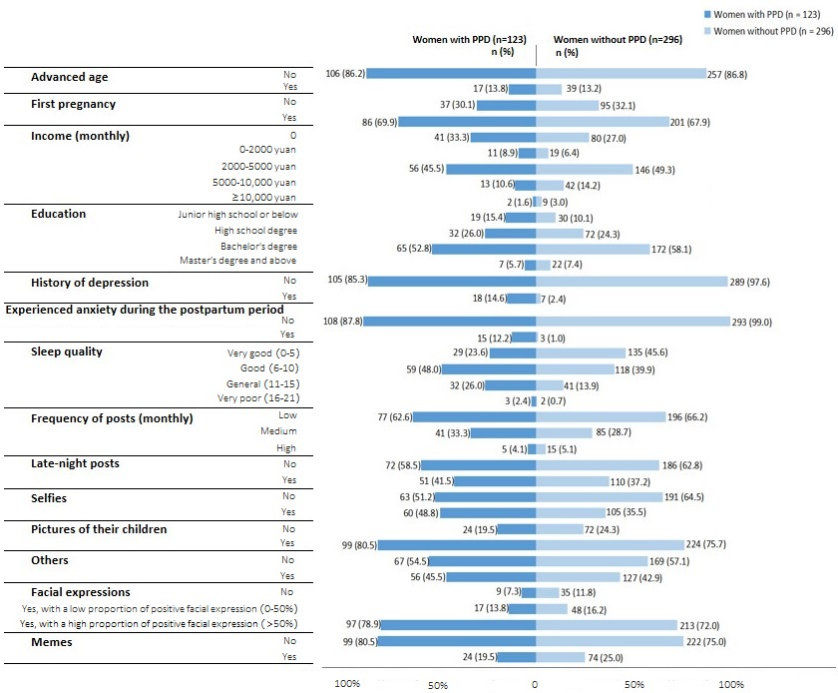
Characteristics of women with or without postpartum depression. PPD: postpartum depression.

We collected a total of 15,647 images and 6609 posts from 419 mothers on WeChat Moments. The demographic characteristics of the sample are shown in Figure 2. The average participant age was 27.6 years (SD 3.8 years). Participants predominantly had a monthly income of 2000-5000 yuan (US $282-705) and had completed undergraduate or college education. The average hue, saturation, and value of the images was 33.9 (SD 10.6), 0.3 (SD 0.1), and 0.6 (SD 0.1), respectively. During the postpartum period, mothers posted an average of 0.89 (SD 5.07) images per day. The incidence of postpartum depression was 29.4% (123/419). Sample comparison analyses were conducted to compare the demographic features of mothers with postpartum depression and the mothers without postpartum depression (Figure 2).

### Image Analysis

#### Content

**Figure 3:**
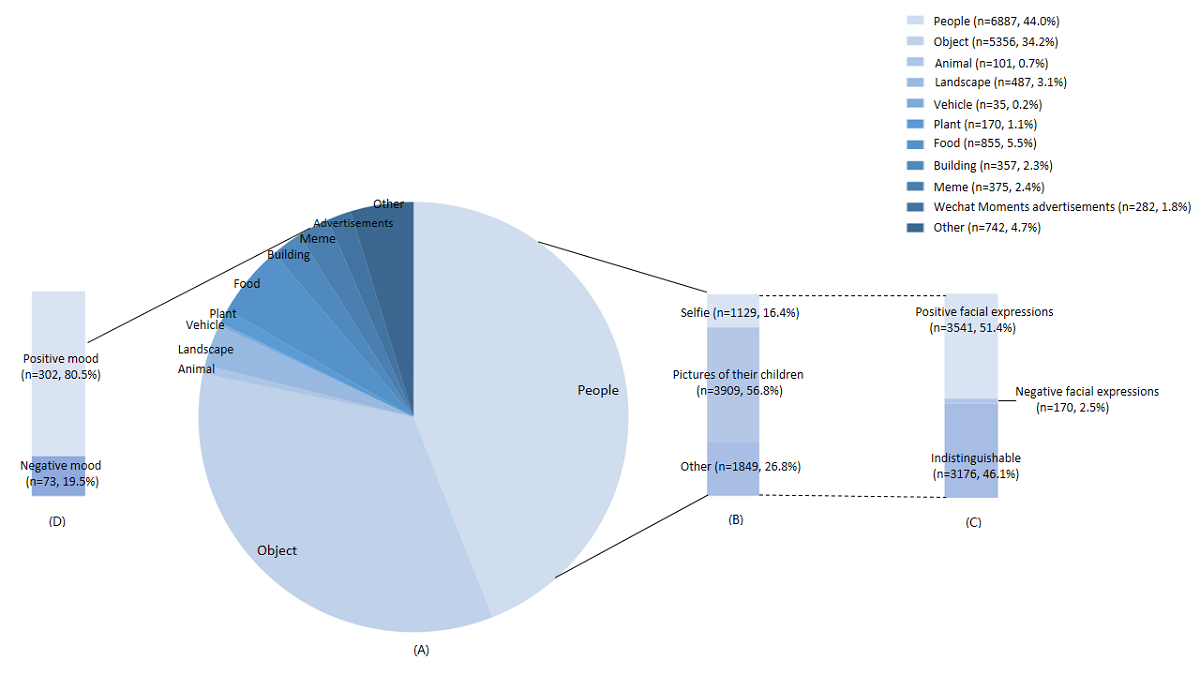
Content and emotions of the images. (A) The number and proportion of each tag type (n=15,647). (B) Content of pictures with people (n=6887). (C) The number and proportion of positive and negative facial expressions (n=6887). (D) The positive and negative moods in the memes posted by the mothers (n=375).

We calculated the number and proportion of each tag type (Figure 3A). Among the images posted by mothers during the postpartum period, the highest proportion was pictures of people (6887/15,647, 44.0%), followed by pictures of objects (5356/15,647, 34.2%).

#### Pictures of People

Pictures of the mothers’ children accounted for the highest proportion (3909/6887, 56.8%) of the pictures of people uploaded by the mothers during the postpartum period (Figure 3B), and most of the people in the pictures had positive facial expressions (3541/6887, 51.4%) (Figure 3C).

#### Memes

Mothers posted more memes depicting a positive mood (302/375, 80.5%) than a negative mood (19.5%) during the postpartum period (Figure 3D).

### Correlation Between Image Features and Postpartum Depression

We used logistic regression to explore the relationship between the image features and the risk of 6-week postpartum depression (Table 1). In this model (*r*^2^ = 0.20), selfies showed statistical significance after controlling for potential confounders. The factors associated with postpartum depression were included in the model as potential confounders. Women who posted selfies in the postpartum period were more likely to have postpartum depression (*P*=.003; odds ratio 2.27, 95% CI 1.33-3.87).

We put the variables of advanced age, first pregnancy, monthly income, education, depression history, anxiety, sleep quality, frequency of posts, late-night posts, colors, facial expressions, selfies, pictures of their children, others, and memes into the logistic regression model and presented the meaningful results in Table 1 (α=.05).

**Table 1:** Logistic regression analysis of the association between image features and postpartum depression risk.

Item		B	*P* value	Odds ratio (95% CI)
Anxiety		2.17	.002	8.71 (2.21-34.39)
**Sleep quality**				
	Very good	—^a^	—	—
	Good	0.90	.001	2.47 (1.42-4.28)
	General	1.25	<.001	3.50 (1.74-7.01)
	Very poor	1.91	.07	6.75 (0.84-54.55)
Selfies		0.82	.003	2.27 (1.33-3.87)

^a^Not available.

## Discussion

### Principal Findings

We found that, during the postpartum period, the mothers in this study tended to post pictures of their children and that the majority of the “people” in the collected pictures were categorized as experiencing positive emotions. However, the mothers who posted selfies during the postpartum period were more likely to have postpartum depression. Therefore, images posted by new mothers on WeChat Moments could be predictive of the mothers’ mental health during the postpartum period.

Pictures of their children accounted for the largest proportion of images posted by the mothers (3909/6887, 56.8%), and 77.1% (323/419) of the mothers posted pictures of their children to WeChat Moments. This result is similar to that of the Bartholomew et al [30] study in which the proportion was 78.6%. Maternal identity confirmation may be the main reason that mothers share pictures of their children during the postpartum period. Johnson [31] suggested that social media was the main social platform mothers used to announce their pregnancies and the births of their babies. Another important reason mothers post pictures of their children may be that they need to strengthen ties with family and friends so that they can obtain more support after childbirth. The Gameiro et al [32] study found that mothers believed they received increased support from their nuclear family and friends in the pre- to postpartum period. Given that adults are more likely to give attention to babies’ faces, many mothers who uploaded photos of their children to social media thought it was “likely” that the photos would be acknowledged (ie, commented on or “liked”) by their social network friends [30]. It is possible that the majority of mothers consider posting pictures of their children as a particularly compelling way to engage with their online friends [30,33]. Accordingly, WeChat Moments may provide mothers with a platform through which to maintain strong ties with, for example, family and close friends. Meanwhile, sharing pictures of their children on WeChat Moments could be a particularly important means for mothers to identify themselves, build and maintain social capital, and find social support.

There were more positive emotions in the facial expressions of the people in the pictures and memes in this study than negative ones. In Chinese tradition, giving birth is a happy life event that should be celebrated by the whole family. Although we assumed that posting on WeChat Moments would reflect the feelings of the mothers in this study more accurately than posts made on other social media due to the WeChat Moment privacy settings, an element of social desirability response bias may have affected the expression of positive emotions. Pressures to portray one’s best self may lead people to display deceptive versions of themselves on social media [28,34]. Social media users tend to present the happiest and most ideal sides of themselves even if these versions do not align with their actual emotions or experiences [35,36]. The findings of this study suggest that even if a new mother shows positive emotions on WeChat Moments, the risk of postpartum depression cannot be ruled out.

The posting of selfies on WeChat Moments seems to indicate the need to pay more attention to possible depressive symptoms in the users, as the women in our study who posted selfies during the postpartum period were more likely to have postpartum depression. This supports the findings of previous studies, which have shown a positive association between selfies and depression [16,28]. One potential explanation for the trend is that mothers posting selfies intend to show an idealized version of themselves, which may cause them to focus on perceived self-deficiencies, thus leading to depression [16,28]. In addition, a study found that women who posted selfies felt more anxious, less confident, and less physically attractive after posting, indicating that selfies may have harmful effects on mental health [16]. Posting selfies may be indicative of mothers’ concerns regarding others’ opinions of their images as well as their desire for more social attention [27,28]. Posting selfies may be one of the things that mothers can do during the “doing the month” period when their social contacts may be weaker. However, excessive concern for others’ opinions, especially those of close friends and family, could negatively impact women’s happiness and increase their risk of postpartum depression [37].

### Limitations

This study had several limitations. First, the convenience sampling method used in this study limited the extrapolation of the results. Second, WeChat Moments allows users to upload pre-processed images, and some women were accustomed to using filters to embellish their images. Our inability to remove the effects of filters on the original images may have led to some bias. However, we believe that filter tones may have reflected some of the users’ emotional states [14]. Third, WeChat has a feature to hide certain posts from certain people, so we cannot be confident that we collected all the mothers’ images on WeChat Moments, even though we asked for all posts to be open to us when we requested informed consent. Fourth, we collected images posted before the EPDS measurement. However, the EPDS measurement reflects symptoms over the 7-day period before the EPDS questionnaire is administered. This means that we could not be certain about the order or cause-and-effect relationship between the images posted and the presence of postpartum depressive symptoms. Longitudinal research is needed to detect whether posting selfies during the postpartum period has a detrimental effect on mothers’ mental health. Lastly, we analyzed the relationship between the mothers’ posted images and postpartum depression but ignored the text accompanying the posts. The text could have helped us understand the content of the images better. We believe that a combined analysis of both text and images could provide more information, and this could be a direction for future research.

### Conclusions

WeChat Moments is an important social media platform for Chinese mothers to share pictures of their newborns. Although mothers tend to post images featuring positive emotions, these pictures may actually mask depression. Particular attention should be given to new mothers who have posted selfies, as they seemed to be at higher risk for postpartum depression.

## Abbreviations

EPDS: Edinburgh Postnatal Depression Scale
